# Supplementary material for: Fiber-based angular demultiplexer using nanoprinted periodic structures on single-mode multicore fibers
Source: Nat Commun. 2025 Mar 7;16:2294. doi: 10.1038/s41467-025-57440-2 (PMC11889240; doi:10.1038/s41467-025-57440-2)
Supplement: Supplementary file 1 — Supplementary Information [file 41467_2025_57440_MOESM1_ESM.pdf]

Supplementary Information: Fiber-based angular demultiplexer using nanoprinted periodic structures on single-mode multicore fibers

Oleh Yermakov[1,2*], Matthias Zeisberger[1], Henrik Schneidewind[1], Adrian Lorenz[1], Torsten Wieduwilt[1], Anka Schwuchow[1], Mohammadhossein Khosravi[1], Tobias Tiess[3], Markus A. Schmidt[1,4,5*]

[1]Department of Fiber Photonics, Leibniz Institute of Photonic Technology, Albert-Einstein-Straße 9, 07745 Jena, Germany.
[2]Department of Computational Physics, V. N. Karazin Kharkiv National University, 4 Svobody Square, 61022 Kharkiv, Ukraine.
[3]Heraeus Comvance, Heraeus Quarzglas GmbH & Co. KG, Heraeusstraße 12, 06803 Bitterfeld-Wolfen, Germany.
[4]Abbe Center of Photonics and Faculty of Physics, Friedrich-Schiller-University Jena, Fürstengraben 1, 07743 Jena, Germany.
[5]Otto Schott Institute of Material Research, Fraunhoferstraße 6 , 07743 Jena, Germany.

*Corresponding author(s). E-mail(s): oe.yermakov@gmail.com; markus-alexander.schmidt@uni-jena.de;

Supplementary Note 1. Properties of bare multicore fiber (MCF)

Beam properties: The seven cores (intercore spacing $\Lambda_{\text{MCF}} = 36$ µm) of the MCF (outer diameter is equal to 135 µm) are designed to resemble the properties of the SMF-28, which is the most widely used SMF. To verify this, the profiles of the modes of two selected cores (center core and one satellite core) were measured optically at $\lambda_0 = 1550$ nm (Laser LDM1, Thorlabs GmbH, Supplementary Fig. 1) and the corresponding NA was determined. Specifically, the mode field diameter (MFD) of the two modes on the fiber surface was measured via imaging, yielding roughly the same value for both cores (MFD $\approx$ 10.3 µm), matching the value of the SMF-28 ($\text{MFD}_{\text{SMF-28}} = 10.4$ µm [1]). The resulting divergence angle and numerical aperture were calculated to be $\theta_{\text{div}} = \arctan\left[2\lambda_0/(\pi\,\text{MFD})\right] \approx 5.5°$ and $NA = \sin\theta_{\text{div}} \approx 0.095$.

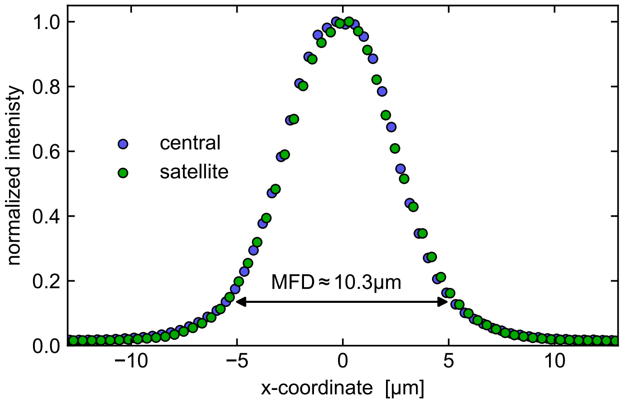

Supplementary Figure 1 Measured intensity distribution (normalized to the maximum) along a selected line on the surface of the single-mode MCF of two exemplary selected cores (blue dots: central core, green dots: satellite core) at $\lambda_0 = 1550$ nm. The mode field diameter (MFD) was determined with respect to the $1/e^2$ level.

Single-mode domain: Another key property of the MCFs used here is that they are single-mode in the spectral domain of interest at around $\lambda_0 = 1550$ nm. This was confirmed by measuring the bending losses on two selected cores (Supplementary Fig. 2). Specifically, a long piece of fiber (length of 200 cm) was bent in a defined manner (bending radius is 1.5 cm) and the light transmission was measured spectrally resolved using the spectrometer Spectro 320D (Instrument Systems). A pronounced maximum of the bending losses was observed in the region around $\lambda \approx 1100$ nm, which can be attributed to the increase in bending-induced losses of higher-order modes. On the long wavelength side of this maximum (yellow area in Supplementary Fig. 2), the cores start to get single-mode so that the fiber supports only one mode per core at the operating wavelength of $\lambda_0 = 1550$ nm.

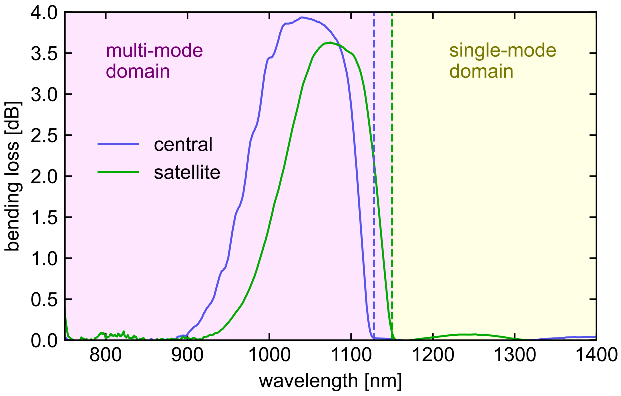

Supplementary Figure 2 Measured bending losses of two selected cores (blue: center core, green: satellite core). The maximum shows the losses caused by a higher-order mode when the fiber is bent. For $\lambda > 1150$ nm the cores of the MCF are single mode (light yellow area), while below multimode guidance is observed (light purple area). The two vertical dashed lines indicate the higher-order mode cut-offs.

Modal attenuation: Another important parameter being key in the context of Fiber Optics is the modal attenuation. For the MCF used in the present work, the loss for the central core and a satellite core were measured using the cut-back method (initial fiber length: 3780.6 m, remaining length after cut-back: 2 m, Supplementary Fig. 3). The resulting distribution of modal losses shows that at the operating wavelength ($\lambda_0 = 1550$ nm), the losses are around $\gamma \approx 1$ dB/km, which do not play a role for the fiber lengths used here (< 10 m).

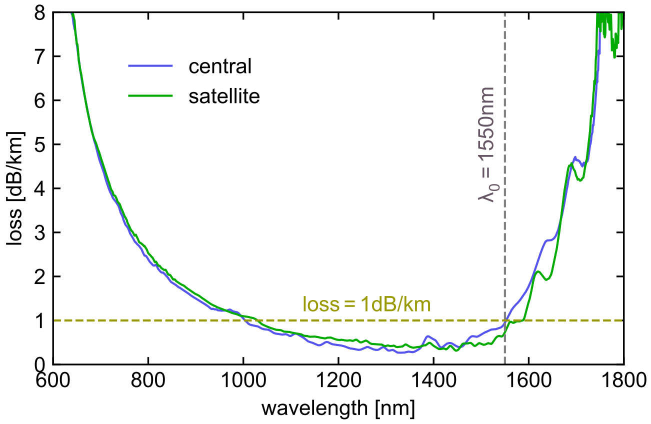

Supplementary Figure 3 Measured spectral distribution of the model attenuation of the fundamental modes in two selected cores of the single-mode MCF (blue: center core, green: satellite core). The vertical gray dashed line shows the operating wavelength ($\lambda_0 = 1550$ nm). The horizontal yellow line shows the loss level of $\gamma = 1$ dB/km.

Intermodal cross-coupling: To quantify the crosstalk of the MCF used, additional experiments were performed. Specifically, to evaluate the modal coupling between the cores (i.e., intercore cross-coupling), broadband light was injected into the central core of the MCF (using a combination of a supercontinuum light source, coupling optics, and spectral diagnostics) and the power transmission of the central core and one of the side cores was measured. This procedure was performed for three bending radii by placing a portion of the MCF in a support plate with customized grooves to create precise bending conditions. Note that the related experimental setup included microscopes at the input and output sides of the MCF to (i) directly observe which core was excited and (ii) collect light from a specific core at the output. To remove the spectral characteristics of the light source and optics, the difference of the power transmission (on a logarithmic dB scale) between the center-center and center-side core configurations ($\Delta t[\text{dB}] = t_{cs}[\text{dB}] - t_{cc}[\text{dB}]$) was calculated (Supplementary Fig. 4(a)). The results show no inter-core cross-coupling up to an extinction ratio of 25 dB over the entire spectral range and especially at the operating wavelength of $\lambda_0 = 1.55$ µm (vertical gray dashed line in Supplementary Fig. 4(a)), which is important for unambiguously correlating angles of incidence and power in the corresponding core. It should be noted that the current dynamic range of this setup is currently limited to about 25 dB, probably due to residual light of the center core entering the objective when the output objective is adjusted to measure one of the side cores. To further confirm the absence of intermodal cross-coupling at the operating wavelength, narrowband light (center wavelength 1550 nm, bandwidth 10 nm) was coupled into the center core and the

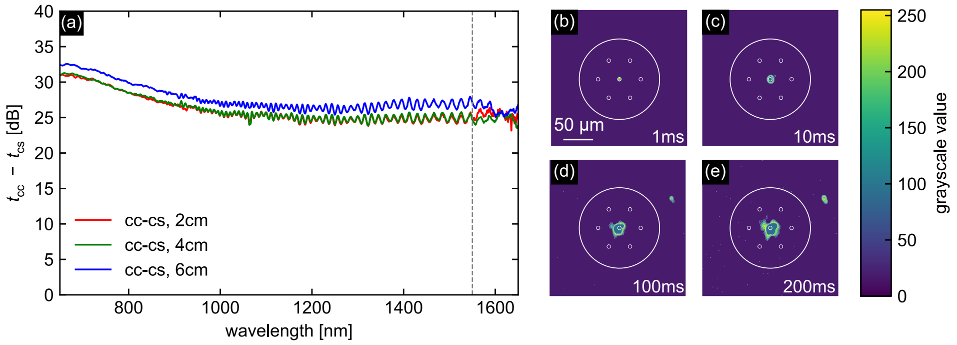

Supplementary Figure 4 (a) Spectral distribution of the transmission difference between the center-center (cc) and center-side (cs) configurations (details in the main text) for three different bend radii (red: 2 cm, green: 4 cm, blue: 6 cm). The vertical gray dashed line refers to the operating wavelength of 1.55 µm. The images on the right (b)-(e) show the measured output intensity distribution when light is injected into the center core at different exposure times of the IR camera used (indicated in the lower right of each image, taken with an ABS-Jena IK1513 camera). The small and large white circles mark the cores positions and the outer diameter of the MCF, respectively. A bandpass filter (FWHM bandwidth: 10 nm, central transmission wavelength: 1550 nm) was inserted into the input beam path to selectively measure the properties at the operation wavelength (indicated by the vertical gray dashed line in (a)). The feature to the right of (d) and (e) is a confirmed imaging artifact caused by reflections within the imaging setup, while the visible deformation of the center beam in (d) and (e) is due to imaging artefacts.

output mode was imaged with an infrared camera (details in the caption of Supplementary Fig. 4). To increase the dynamic range of the camera, grey scale images were taken at different exposure times (from 1 ms to 200 ms), increasing the initial 8-bit dynamic range of a single image from 23 dB to 46 dB (Supplementary Figs. 4(b)-4(e)). This procedure results in strong saturation in the middle of the images dominated by light from the central core at high exposure times (Supplementary Figs. 4(d)-4(e)), while none of the images show any sign of intensity in the side cores, confirming the absence of intermodal cross-coupling.

Supplementary Note 2. Description of nanostructure designing optimization procedure

The individual steps of the optimisation process are described in detail below.

Step 1: determination of pitch.

The first step allows defining the pitch and results from the relationship between the wave number of the grating $G = 2\pi/\Lambda$ and the in-plane wave vectors of the incident k_x and diffracted $k_{||}^m$ waves: $k_{||}^m = k_x + mG$. The highest coupling is achieved when the diffracted wave propagates along the fiber axis, i.e. $k_{||}^m = 0$, leading to the condition $k_x = -mG$, in other terms $\sin\theta_m/\lambda_0 = -m/\Lambda$. In the present work, the relevant order of diffraction is $m = -1$, leading to $\sin\theta_m = \lambda_0/\Lambda$.

Step 2: derivation of first ring position.

The first step relies on analyzing the overlap integral between the electric field components of fiber mode and diffracted waves, which is used to calculate the coupling efficiency [2]. The associated integrand is proportional to the expression $f(r) = J_2(k_x r) - J_0(k_x r)$, which results from the decomposition of the incident plane wave into cylindrical coordinates according to the Jacobi-Anger expansion [3]. Note that $k_x = 2\pi\sin\theta_m/\lambda_0$ and $J_m(x)$ is the first order Bessel function of order m. The center of the first ring should therefore be at $r = r_0$, where r_0 is related to the maximum of $\max f(r) = f(r_0)$ and leads to the highest coupling efficiency of a plane wave into the guided fiber mode for the incident angle m. We have empirically found that the angular dependence of the first ring position may be expressed as $r_0(\theta_m) = 2\Lambda(\theta_m)/\pi = 2\lambda_0/(\pi\sin\theta_m)$.

Step 3: determination of geometric ring parameters.

The idea behind the determination of the height of the grating elements is based on the suppression of a phase delay caused by the nanostructure, which requires that the phase difference of the waves inside and outside the polymer be a multiple of π, namely $h = \lambda_0/(2\Delta n)$ ($\Delta n = n_p - n_{air}$: difference between the refractive indices of polymer and air). This condition follows from the assumption that the phase delay between the waves inside and outside the polymer should be equal to π. The width of the elements is set to $w = \Lambda/2$, following the idea that the parts of electromagnetic power passing through polymer and air are equal.

Step 4: numerical optimization for fine adjustment.

In the final step, the structure is optimized by numerical analysis using finite-element method modelling (COMSOL Multiphysics) for different angles of incidence. The details of the numerical procedure are described in Ref. [2]. The previously determined lattice parameters are used as initial parameters to maximize the coupling efficiency for selected angles of incidence. Interestingly, it is found that the parameters determined in the first two steps already result in a near-optimal coupling efficiency.

Supplementary Note 3. Example of optimization procedure

The optimization procedure aims to maximize the light coupling to the fiber modes is described in detail in the following. The idea of the procedure is to find a design for the polymer ring structure ($n_p = 1.534$) that maximizes in-coupling efficiency, shown here on the example of $\theta_m = 40°$ (core diameter is 6.1 µm, core (cladding) refractive indices are 1.45 (1.444), $\lambda_0 = 1.55$ µm). A detailed step-by-step instruction and the corresponding results are shown in Supplementary Fig. 5. Following the derived procedure, we first find the lattice constant equal to $\Lambda = \lambda_0 / \sin(40°) = 2.41$ µm. In the next, the first ring position is defined as $r_0 = 1.53$ µm ($k_x r_0 = 3.99$) according to step 2 [vertical dashed line in Supplementary Figs. 5(a)-5(d)]. One can notice that

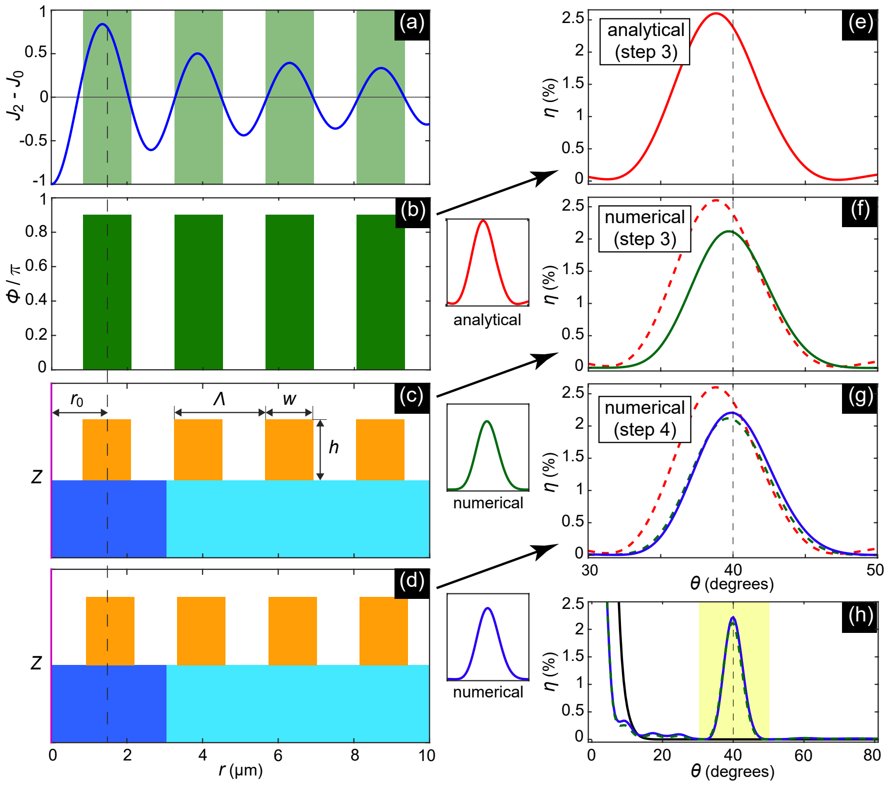

Supplementary Figure 5 Details of the optimization procedure, demonstrated here on the example of $\theta_m = 40°$ (described in all plots by the vertical black dashed line, $\lambda_0 = 1.55$ µm). (a) Definition of r_0 by the function $f(r) = J_2(k_x r) - J_0(k_x r)$. (b) Radial dependence of the phase factor describing the axial-symmetric structure $\Phi(r)$. (c,d) Schematic cross-section view of the ring arrangements for (c) the analytic-based and (d) numerically optimized designs corresponding to the steps 1–3 and 4 of the optimization procedure. (e-h) Angular distribution of the in-coupling efficiency using (e) the phase factor approach (red line), (f,h) the analytic-based design (green lines) and (g,h) the numerically optimized design (blue lines) calculated (e-g) in the vicinity of $\theta_m = 40°$ and (h) over the entire angular range. The black line in (h) corresponds to the bare fiber.

the value of r_0 nearly corresponds to the first peak of the Bessels-related function $f(r)$ [Supplementary Fig. 5(a)]. Finally, we determine the geometric rings parameters using the equations from step 3 (w = 1.205 µm, h = 1.45 µm). Note that according to the analytical model reported in Ref. [2], the axially symmetric structure can be replaced by a step-wide radial phase distribution $\Phi(r)$ corresponding to the ring positions [Supplementary Fig. 5(b), resulting coupling efficiency shown in Supplementary Fig. 5(e)]. The cross-section view of the analytic-based design obtained using steps 1–3 is shown in Supplementary Fig. 5(c), the corresponding in-coupling efficiency is shown in Supplementary Fig. 5(f). One can notice that the local angular maximum is slightly shifted by around $-0.4°$ relative to θ_m. The following numerical optimization (step 4) leads to the final design with the parameters r_0 = 1.5 µm, Λ = 2.41 µm, w = 1.21 µm, h = 1.69 µm [the cross-section view is shown in Supplementary Fig. 5(d)], the corresponding coupling efficiency is shown in Supplementary Fig. 5(g). One can notice that the angular position of the peak now precisely matches $\theta_m = 40°$ with its amplitude being 1.05 times higher than for the analytic-based design. For comparison, the in-coupling efficiency obtained after step 3 and step 4 across the entire angular range from 0° to 90° is shown in Supplementary Fig. 5(h).

The comparison between the analytic-based design (step 3) and the final numerically optimized design (step 4) in Supplementary Table 1 proves the relevance of a purely theoretical approach with a relative difference not exceeding 5%. Note that the local maxima angles for the analytic-based design may be shifted from 0.3° up to 1.2° from the desired values.

	step 1	step 2	step 3 (analytic-based design)	step 4 (optimized design)	relative difference
target	definition of lattice constant	definition of position of first ring	definition of rings parameters	parameters fine tuning	–
approach	$\Lambda = \lambda_0/\sin\theta_m$	$r_0 = 2\Lambda/\pi$	$w = \Lambda/2$, $h = \lambda_0/(2\Delta n)$	final numerical optimization	–
Λ	2.41 µm	2.41 µm	2.41 µm	2.41 µm	0
r_0	–	1.53 µm	1.53 µm	1.5 µm	2%
w	–	–	1.205 µm	1.21 µm	0.4%
h	–	–	1.45 µm	1.69 µm	14.2%
θ_{-1}	–	–	39.7°	40°	0.8%
$\eta(\theta_{-1})$	–	–	2.1%	2.2%	4.6%

Supplementary Table 1 Summary of the individual optimization steps for the example discussed in the main text ($\theta_{-1} = 40°$). The rightmost column shows the relative difference between the analytic-based and numerically optimized designs, i.e., steps 3 and 4.

Supplementary Note 4. Comparison between analytic-based and numerically optimized designs

Using the same procedure, we obtain optimized designs for the different local maxima angles. A full comparison between the analytic-based and optimized designs with the corresponding relative differences is given in Supplementary Tab. 2.

core ID	#1	#2	#3	#4	#5	#6	#7
pitch (Λ), µm [analytic-based design]	4.53	3.1	2.41	2.02	1.79	1.65	1.57
pitch (Λ), µm [optimized design]	4.4	3	2.41	2.05	1.78	1.65	1.54
relative difference for pitch (δ_Λ), %	3	3.3	0	1.5	0.6	0	2
1st ring center (r_0), µm [analytic-based design]	2.88	1.97	1.53	1.29	1.14	1.05	1
1st ring center (r_0), µm [optimized design]	3	2	1.5	1.2	1.1	1.05	0.95
relative difference for 1st ring center (δ_{r_0}), %	4	1.5	2	7.5	3.6	0	5.3
ring width (w), µm [analytic-based design]	2.265	1.55	1.205	1.01	0.895	0.825	0.785
ring width (w), µm [optimized design]	2.2	1.5	1.21	1.03	0.89	0.83	0.77
relative difference for ring width (δ_w), %	3	3.3	0.4	1.9	0.6	0.6	2
ring height (h), µm [analytic-based design]	1.45	1.45	1.45	1.45	1.45	1.45	1.45
ring height (h), µm [optimized design]	1.1	1.5	1.69	1.44	1.25	1.15	1.23
relative difference for ring height (δ_h), %	31.8	3.3	14.2	0.7	16	26.1	17.9
local max. angle (θ_m), ° [analytic-based design]	19.7	29.7	39.7	49.3	58.9	69.9	76
local max. angle (θ_m), ° [optimized design]	20	30	40	50	60	70	80
relative difference for local max. angle (δ_{θ_m}), %	1.5	1	0.8	1.4	1.8	0.1	5
$\eta(\theta_m)$, % [analytic-based design]	4.3	2.5	2.1	1.5	1.1	0.9	0.8
$\eta(\theta_m)$ [optimized design]	3.8	2.6	2.2	1.4	1	0.9	0.7
relative difference for in-coupling ($\delta_{\eta_{\max}}$), %	-10.5	3.9	4.6	-7.1	-10	0	-14.3

Supplementary Table 2 Comparison between the analytic-based (steps 1–3) and numerically optimized nanostructure designs ($\lambda_0 = 1.55$ µm).

The relative difference for different parameters calculated here was defined as

$$\delta_\epsilon = \frac{|\epsilon_{\text{opt}} - \epsilon_{\text{an}}|}{\epsilon_{\text{opt}}}, \tag{1}$$

where ϵ refers to the corresponding values $[\Lambda, r_0, w, h, \theta_m$ or $\eta(\theta_m)]$ for the analytic-based and optimized designs (indices 'an' and 'opt', respectively). Please note that for the $\delta_{\eta_{\max}}$ the absolute value operator was eliminated, so the sign minus in the relative difference means the higher values of the in-coupling efficiencies for the analytic-based design.

The analytically and numerically obtained normalized in-coupling efficiencies are shown in Supplementary Fig. 6. A good correspondence between analytical and numerical calculation, as well as between the analytic-based and numerically optimized designs is found. The final optimized geometric parameters are listed in Table 2, while their simulated and measured in-coupling efficiency spectra are shown in Figs. 3(a) and 5(a). It is important to note that the in-coupling efficiencies for a bare fiber are

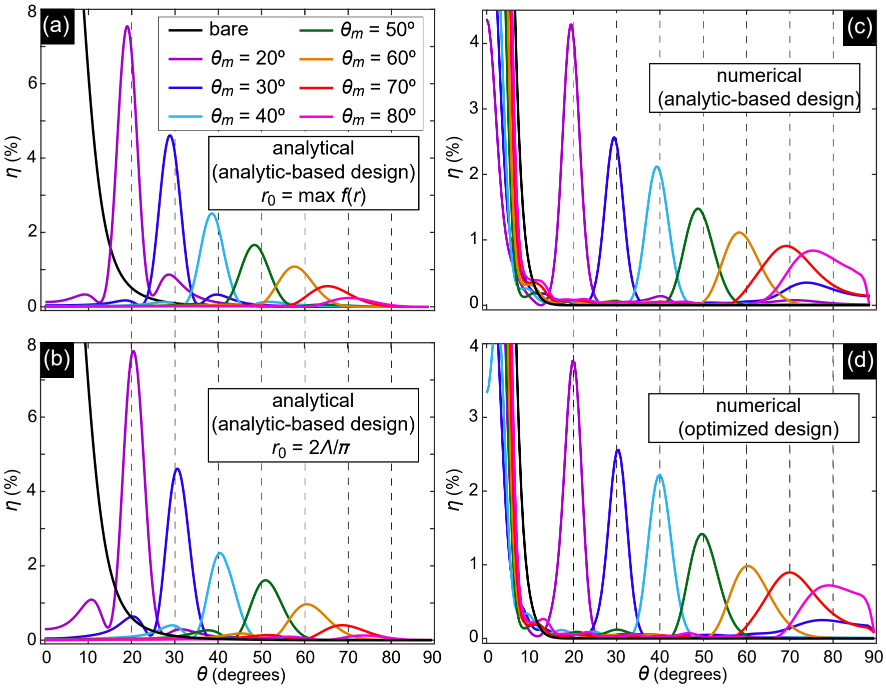

Supplementary Figure 6 Angular distribution of coupling efficiencies for the seven designs of axially symmetric structures with different local maxima ($20^\circ < \theta_m < 80^\circ$, in steps of $\Delta\theta = 10^\circ$, shown by the vertical gray dashed lines, $\lambda_0 = 1.55$ µm). Here, (a,b) show the angular distributions calculated analytically with different approach for the first ring position: (a) $r_0 = \max[J_2(k_x r) - J_0(k_x r)]$, (b) $r_0 = 2\Lambda/\pi$, while (c,d) show the angular distributions calculated numerically. The cases (a-c) and (d) are calculated for the analytic-based (steps 1–3) and optimized (step 4) designs following the optimization procedure.

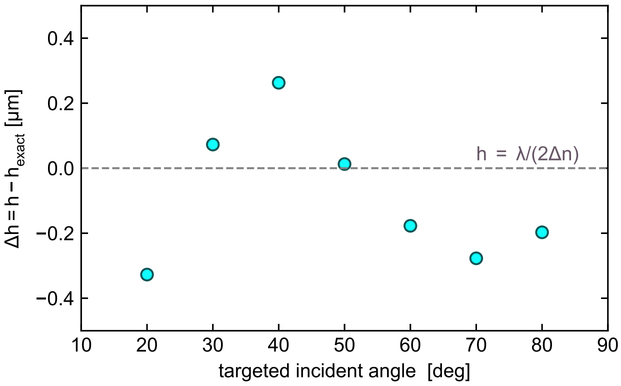

Supplementary Figure 7 Dependence of the numerically optimized value for the grating height on the target angle of incidence. Note that the difference relative to the value specified in step 3 of the optimization procedure is plotted (horizontal gray dashed line).

below the noise value for angles $\theta > 20°$, while the simulated and measured values reach the percentage level.

The height distribution of the optimized grating structures (step 4) is shown in Supplementary Fig. 7 for the different target incidence angles. A nonlinear behavior can be clearly seen, with the points oscillating around the value $h = \lambda_0/(2\Delta n)$ defined in step 3 (analytic-based design). Supplementary Figure 8 shows the angular dependence of the in-coupling efficiency in the vicinity of local maximum for different heights of the grating #3 from Tab. 1 ($\theta_{-1} = 40°$). In fact, there is practically no change in the angular position of the maximum, with a slight change in the amplitude of the coupling efficiency by less than 0.2% for a decrease or increase in height of 300 nm.

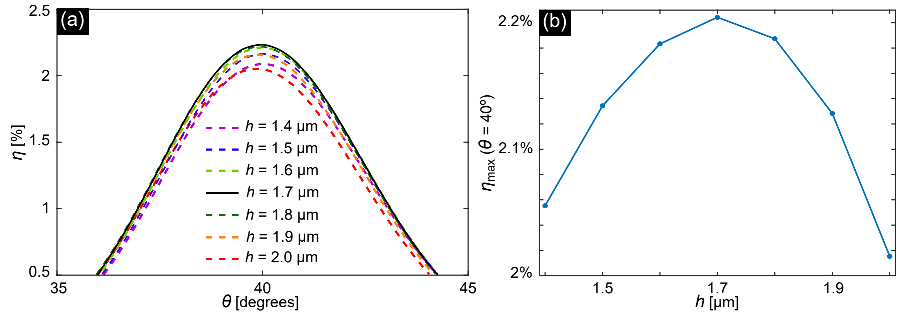

Supplementary Figure 8 (a) Angular dependence of the coupling efficiency for a nanostructure-enhanced single-mode fiber with a design angle of incidence of $\theta_{-1} = 40°$ (grating #3 from Tab. 1) for varying grating element heights. (b) Local maximum of coupling efficiency as a function of element height.

Supplementary Note 5. Selectivity and spectral dependence of fiber-based device

Selectivity. To prove the selectivity of the fiber-based device developed, we derive the procedure including (i) identifying the core with the highest power, which corresponds to one of the colored curves in Fig. 3(a); and (ii) comparing the power levels of modes of several related cores in order to unambiguously determine the angle of incidence. We examine the simulated coupling efficiencies at $\lambda_0 = 1.55$ µm for three closely spaced angles around an incident angle of 40° ($\theta = 38°, 40°, 42°$). The corresponding values of the coupling efficiencies of the gratings with the design angles 30°, 40° and 50° – $\eta_{30°}$, $\eta_{40°}$ and $\eta_{50°}$ – are given in Supplementary Tab. 3 as well as the ratios between these coupling efficiencies ($\eta_{30°}/\eta_{40°}$ and $\eta_{50°}/\eta_{40°}$). One can notice that the values of the coupling efficiencies for the three angles of incidence are significantly different and well separated, demonstrating that the concept allows unambiguous identification of the angle of incidence.

θ	38°	40°	42°
$\eta_{30°}$ [%]	0.0053	0.0282	0.0407
$\eta_{40°}$ [%]	2.2168	2.9151	2.1446
$\eta_{50°}$ [%]	0.0767	0.0433	0.0031
$\eta_{30°}/\eta_{40°}$ [%]	0.0024	0.0097	0.0190
$\eta_{50°}/\eta_{40°}$ [%]	0.0346	0.0149	0.0015

Supplementary Table 3 Example of selected simulated coupling efficiencies, taken from Fig. 3(a), for three closely spaced angles of incidence ($\theta = 38°, 40°, 42°$), showing the selectivity of the device. The first three rows show the coupling efficiencies for three different cores (i.e., nanostructures) resulting from the curves shown in Fig. 3(a) ($\eta_{30°}$: blue curve, $\eta_{40°}$: cyan curve, $\eta_{50°}$: green curve). The two lower rows show the corresponding ratios of the coupling coefficients (top: $\eta_{30°}/\eta_{40°}$, bottom: $\eta_{50°}/\eta_{40°}$).

Spectral dependence. We analyze the effect of spectral variations on both the diffraction angle and the collection efficiency within the telecom C-band (from 1530 to 1565 nm). Specifically, the coupling efficiency was calculated for the nanostructure-enhanced multicore fiber that has a design angle of $\theta = 40°$ at 1550 nm (grating #3 in Tab. 1) in the vicinity of this angle for various wavelengths between 1530 nm and 1565 nm [Supplementary Figure 9(a)]. The results show a minor change in the characteristics, with a negligible variation in the amplitude of the coupling efficiency and a slight spectral shift in the maximum coupling angle $\theta_{\max}$. To quantify this dependence, the maximum angle was plotted as a function of wavelength [Supplementary Figure 9(b)], showing a linear relationship with a slope of $\theta_{\max}/d\lambda = 0.02°/\text{nm}$.

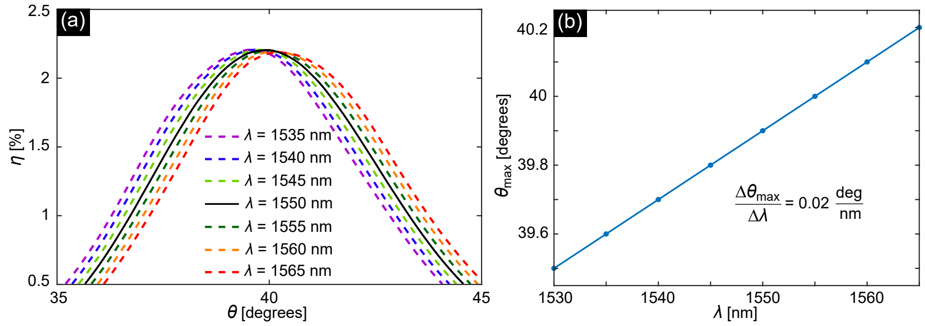

Supplementary Figure 9 Simulated impact of the wavelength dependence on the coupling efficiency. (a) Angular dependence of the coupling efficiency for the grating with a design angle of $\theta_{-1} = 40^\circ$ (grating #3 from Tab. 1) for different wavelengths within the telecom C-band (indicated by different colors). (b) Angle of maximum coupling efficiency as a function of wavelength within the C-band. The resulting slope of this linear dependence is $\Delta\theta_{\mathrm{max}}/\Delta\lambda = 0.02^\circ/\mathrm{nm}$.

Thus, the developed fiber-based device demonstrates high angular and spectral selectivity.

Supplementary Note 6. Implementation and characterization

3D nanoprinting process. The direct laser writing process used to 3D nanoprint polymeric nanostructures on the endface of the multicore fibers is schematically shown in Supplementary Fig. 10 and consists of the following steps:

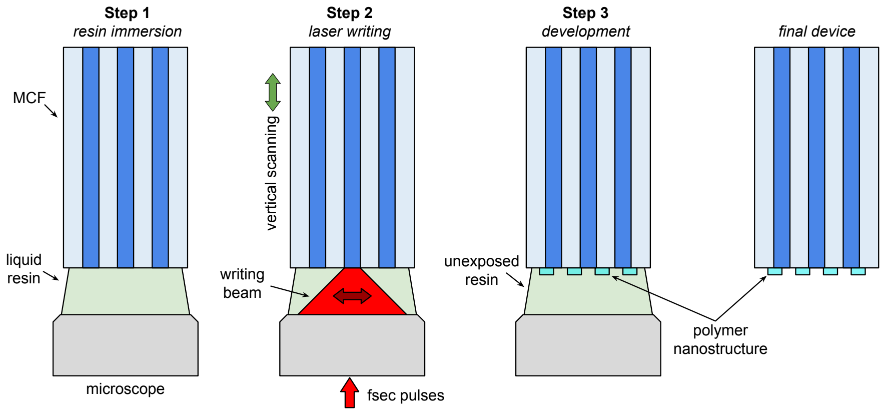

Supplementary Figure 10 Visualization of the individual steps of the 3D nanoprinting process used in this work to create polymeric nanostructures on the end face of multicore fiber (MCF).

Step 1. Resin immersion: The printer, equipped with an inverted microscope (objective pointing upwards), is used for both laser printing and imaging. First, the resin (monomer, IP-dip 2) is applied to the objective and the fiber is fixed in a holder above it with the end facing down. The objective then moves upward, dipping the fiber into the resin and stopping when the fiber reaches the focal distance.

Step 2. Laser writing: The polymer is cross-linked at desired locations using two-photon polymerization (2PP) with a moving femtosecond laser focus. Note that the 2PP process is essential to achieve nanoscale dimensions with direct laser writing due

fiber-based devices	$\eta_{\text{sim}}(\theta = 0°)$	$\eta_{\text{exp}}(\theta = 0°)$
bare fiber	1	1
nanostructure #1 ($\theta_{-1} = 20°$)	0.258	0.305
nanostructure #2 ($\theta_{-1} = 30°$)	0.080	0.118
nanostructure #3 ($\theta_{-1} = 40°$)	0.044	0.143
nanostructure #4 ($\theta_{-1} = 50°$)	0.097	0.197
nanostructure #5 ($\theta_{-1} = 60°$)	0.257	0.360
nanostructure #6 ($\theta_{-1} = 70°$)	0.380	0.446
nanostructure #7 ($\theta_{-1} = 80°$)	0.390	0.424

Supplementary Table 4 Summary of simulated and measured coupling efficiency of the various geometries for normal incidence (details of corresponding geometric parameters can be found in Tab. 1 of the main text).

to the small polymerization volume of 2PP. The laser focus is moved relative to the fiber end face via scanning mirrors in the horizontal plane and by a piezo element in the vertical direction, allowing polymerization along its path and ultimately forming the nanostructures.

Step 3. Development: Finally, the structure is developed by removing the unexposed resin with selected solvents (see the Methods section for details), yielding a sample that can be directly used in the optical experiments.

The nanoprinted polymer structures reduce the coupling efficiency at normal incidence, indicating that the device is designed to operate predominantly in the range of non-normal incidence (Supplementary Tab. 4).

Optical characterization. To measure the in-coupling efficiency, collimated light (IR-SLED, $\lambda_0 = 1550$ nm) was directed to nanostructure at various incident angles and the light output from the opposite end of the fiber was imaged on a camera (a schematic drawing of the setup, including a photographic image is shown in Supplementary Fig. 11). The incident light was collimated using a fiber coupled collimator (Thorlabs F230FC-1550, divergence $< 0.2°$) and then linearly polarized (power at sample is 10.5 mW). To achieve angle-dependent coupling, the fiber was mounted on a rotary stage using a high-precision XYZ stage (rotation accuracy is about 0.5°), and the fiber end face with the dielectric structure was precisely aligned with the axis of rotation. Note that the collimated beam was sufficiently large to uniformly illuminate the entire fiber end face. The output light was imaged onto an infrared camera (resolution: 320×256 pixels, pixel size: 30 µm) via a 60×/0.75 objective. Since the intensity changes significantly between individual cores and over the angular range of interest (in some cases by orders of magnitude), a separate image was recorded for each core with an angular increment of 1°. The exposure time of each image was adjusted to maximize the signal on the camera sensor without causing oversaturation. In addition, different neutral-density filters were used to adjust the intensity. Details of the measurement procedure including a visualization can be found in Supplementary Fig. 12.

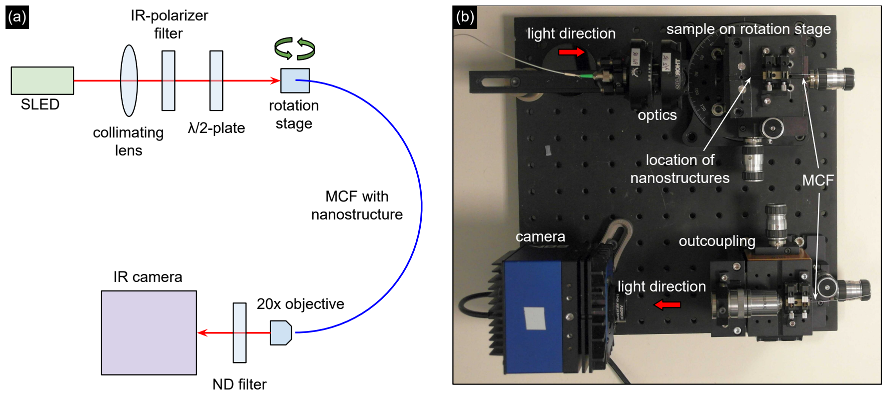

Supplementary Figure 11 Visualization of the experimental setup used to measure the light coupling to different fiber cores. (a) Schematic diagram showing all relevant components (SLED: superluminescent diode, IR: infrared, $\lambda/2$-plate: half-wave plate, ND filter: neutral-density filter, MCF: multicore fiber). (b) Photographic top view of the setup with key components labeled.

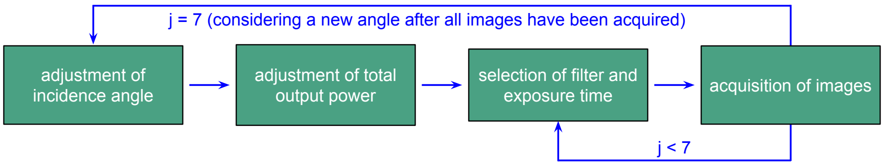

Supplementary Figure 12 Visualization of the measurement procedure for the acquisition of the power distribution over the cores at the output of the nanostructure-empowered MCF.

Amplification factor. The variation in brightness of the images shown in Fig. 6 is due to the different camera settings used during image acquisition. Due to the relatively limited dynamic range of the camera used (8-bit), multiple images were taken for each angle of incidence to compensate for either low intensity or image saturation. The corresponding gain factors for all images relative to the image at normal incidence ($\theta = 0°$) are given in Supplementary Table 5.

incident angle	optimized core (number refers to the respective design angle)	gain factor
0°	30°	1
10°	30°	113
20°	20°	3
25°	20°	227
30°	30°	6.2
35°	30°	0
40°	40°	8.1
45°	50°	38
50°	50°	11.3
55°	60°	47
60°	60°	20
65°	70°	47
70°	70°	47
75°	80°	113
80°	80°	151

Supplementary Table 5 Gain factors used for the images shown in Fig. 6 to compensate for either low intensity or image saturation. Note that the middle column indicates the core for which the image was optimized. In this context, these cores are specified by the design angle of incidence. For example, a value of 50° of the middle column refers to the core in the lower right corner shown in the images of Fig. 6.

Supplementary Note 7. Intensity angular distribution for conventional diffraction grating

The intensity distribution of a transmission diffraction grating (period: Λ, slit width: $\Lambda/2$) can be analyzed using Fraunhofer diffraction principles when illuminated by a monochromatic plane wave [4]. The equation for the intensity $I(\theta)$ at a diffraction angle θ is given by

$$I(\theta) = I_0 \left(\frac{\sin\beta}{\beta}\right)^2 \left(\frac{\sin N\alpha}{\sin\alpha}\right)^2, \tag{2}$$

where I_0 is the incident light intensity, N is the number of slits considered, $\alpha = (\pi\Lambda\sin\theta)/\lambda_0$ and $\beta = \alpha/2$ are the phase difference between waves from adjacent slits and across a single slit. Note that the single-slit term $(\sin\beta/\beta)^2$ modulates the intensity per slit width, while the multi-slit term $(\sin N\alpha/\sin\alpha)^2$ produces sharp peaks at certain angles corresponding to integer multiples of λ_0/Λ, which denote the different diffraction orders. This pattern has bright maxima separated by dark minima, which is critical for applications such as spectral analysis.

As an example, the intensity distribution as a function of diffraction angle was calculated for the case of $\lambda_0 = 1550$ nm and $N = 10$ for four pitches (Supplementary Fig. 13). Clearly visible are the 0th-diffraction order at $\theta = 0°$ and the $\pm1^{\text{st}}$-diffraction orders on both sides of the central maximum. Note that these orders cover larger angular intervals for smaller values of Λ, i.e. for larger diffraction angles.

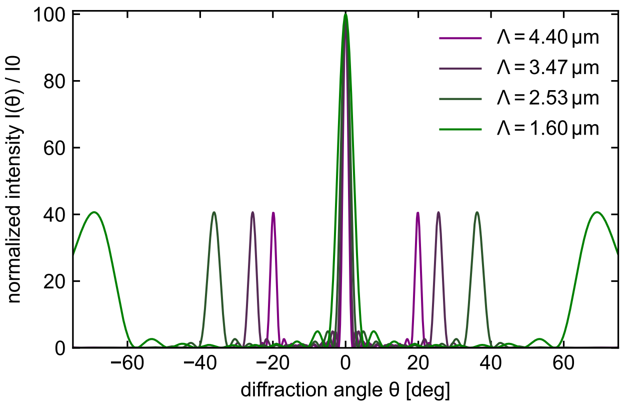

Supplementary Figure 13 Normalized intensity (in %) distributions of the transmission grating as a function of diffraction angle for four different periods (i.e., values of a pitch, $\lambda_0 = 1.55$ µm): $\Lambda = 4.4$ µm (magenta line), $\Lambda = 3.47$ µm (dark-purple line), $\Lambda = 2.43$ µm (dark-green line) and $\Lambda = 1.6$ µm (light-green line).

Supplementary Note 8. Comparison of fiber-based devices performance and fabrication methods

Supplementary Table 6 shows a comparison of the device presented in this work with devices from previous studies in terms of nanostructure design, fabrication methods, fiber types, and mode characteristics. Previous studies have solely focused on implementing various configurations of nanostructures on single-core fibers, such as plasmonic nanodot gratings [5], SiN annular gratings [2], both fabricated by modified electron beam lithography, and complex periodic ring nanostructures by direct laser writing [6, 7]. In contrast, this study is the first to fabricate annular gratings directly on a custom-designed multicore fiber, integrating seven single-mode cores without modal crosstalk.

	[5]	[2]	[6]	[7]	this work
nanostructure	plasmonic nanodot grating	SiN annular grating	polymer annular grating	polymer annular grating	polymer annular grating
lithographic method	modified e-beam	modified e-beam	direct laser writing	direct laser writing	direct laser writing
mode characteristics	single-mode	single-mode	single-mode	multimode	single-mode
No. cores	1	1	1	1	7
single-core / total coupling efficiency (at 40°)	0.02%	0.006%	1.8% (single-pitch structure #1), 0.06% (aperiodic structure)	6.1%	1.4% / 1.5%
single-core / total coupling efficiency (at 70°)	0.002%	0.2%	0.02% (single-pitch structure #3), 0.03% (aperiodic structure)	0.6%	0.25% / 0.4%
capability for angle demultiplexing	no	no	no	no	yes

Supplementary Table 6 Comparison of the key performance indicators of the presented device with those reported in the literature.

Previous studies have reported much lower coupling efficiencies at an incident angle of 40°. For example, the plasmonic nanodot grating by N. Wang et al. [5] achieved only 0.02%, while the SiN ring grating by O. Yermakov et al. [2] reached 0.006%. In comparison, the device in this study achieved a coupling efficiency of 1.4% to 1.5%, a significant improvement over previous designs for a single-mode fiber design, demonstrating improved collection capabilities for off-axis light. Notably, while the multimode fiber system listed in the table achieved even higher coupling efficiencies (up to 6.1%) [7], the larger core and multimodeness do not allow angular multiplexing and may have practical disadvantages compared to the single-mode fibers used in this

work. A similar conclusion can be drawn by comparing the values at an incident angle of 70°.

This comparison highlights the technical advancement achieved in this study, where the multi-core fiber design and optimized nanostructures enable superior light coupling efficiency, particularly at high angles of incident, outperforming previous single-core fiber designs.

Supplementary References

[1] Corning: Data sheet of single mode fiber SMF-28. https://www.corning.com/

[2] Yermakov, O., Schneidewind, H., Hübner, U., Wieduwilt, T., Zeisberger, M., Bogdanov, A., Kivshar, Y., Schmidt, M.A.: Nanostructure-empowered efficient coupling of light into optical fibers at extraordinarily large angles. ACS Photonics **7**(10), 2834–2841 (2020)

[3] Andrews, G.E., Askey, R., Roy, R.: Special Functions vol. 71. Cambridge University Press, Cambridge (1999)

[4] Saleh, B.E., Teich, M.C.: Fundamentals of Photonics. John Wiley & Sons, New York (2019)

[5] Wang, N., Zeisberger, M., Hübner, U., Schmidt, M.A.: Boosting light collection efficiency of optical fibers using metallic nanostructures. ACS Photonics **6**(3), 691–698 (2019)

[6] Yermakov, O., Zeisberger, M., Schneidewind, H., Kim, J., Bogdanov, A., Kivshar, Y., Schmidt, M.A.: Advanced fiber in-coupling through nanoprinted axially symmetric structures. Appl. Phys. Rev. **10**(1), 011401 (2023)

[7] Zeisberger, M., Schneidewind, H., Wieduwilt, T., Yermakov, O., Schmidt, M.A.: Nanoprinted microstructure-assisted light incoupling into high-numerical aperture multimode fibers. Opt. Lett. **49**(8), 1872–1875 (2024)
