# Supplementary material for: Fiber-based angular demultiplexer using nanoprinted periodic structures on single-mode multicore fibers
Source: Nat Commun. 2025 Mar 7;16:2294. doi: 10.1038/s41467-025-57440-2 (PMC11889240; doi:10.1038/s41467-025-57440-2)
Supplement: Supplementary file 2 — Description of Additional Supplementary Files [file 41467_2025_57440_MOESM2_ESM.docx]

**Description of Additional Supplementary Files**

**Supplementary Movie 1**: Measured images of the output intensity of the nanostructured-enhanced multicore fiber (white color corresponds to high intensity) for the different angles of incidence (green numbers in the top left corner).
